# Orthopedic Dermatopathies: Skin Manifestations in Orthopedic Conditions

**DOI:** 10.26502/josm.511500157

**Published:** 2024-08-30

**Authors:** Tony Eskandar, Fihr Chaudhary, Devendra K Agrawal

**Affiliations:** Department of Translational Research, College of Osteopathic Medicine of the Pacific, Western University of Health Sciences, Pomona, California USA

**Keywords:** Brachioradial Pruritus, Dermatopathy, Fibromatosis, Ganglion cyst, Koebner phenomenon, Linear Lichen Planus, Metal allergies, Muscle herniation, Neuropathic itch, Nostalgia Paresthetica, Orthopedic diseases, Osteomyelitis, Psoriatic arthritis, Vitiligo

## Abstract

Orthopedic diseases often present with dermatological symptoms that require prompt identification for appropriate treatment. Understanding these dermatologic presentations is crucial for accurate diagnosis and effective management. This article critically reviewed the dermatological manifestations observed in general and regional pathologies, followed by treatment-related manifestations. An extensive literature search was performed and limited to manifestations in orthopedic disease, excluding those pertaining to infection or syndromes. Case reports and case series documenting unusual and rare dermatologic presentations of orthopedic conditions were examined, providing novel perspectives on both prevalent and uncommon illnesses. The identified pathologies are discussed in detail, including their clinical features and diagnosis, while treatment approach varies depending on the severity of the condition, ranging from self-care to surgical intervention. The findings emphasize the need for interdisciplinary collaboration and highlight the importance of careful diagnosis and appropriate management to eliminate unnecessary approaches and ensure optimal outcomes for patients with orthopedic diseases and dermatologic symptoms.

## 1. Introduction

Orthopedic diseases are a prevalent issue, can arise from either environmental circumstances or genetic predisposition. Rheumatoid arthritis is a chronic illness characterized by pain, inflammation, and rigidity in the feet, hands, and wrists. Osteoarthritis is a persistent joint disorder that impacts the knees, hips, back, and neck, resulting in discomfort and friction between bones. The prevalence of this condition is highest among individuals aged 65 years and above, and it can be influenced by factors such as joint traumas, genetic predisposition, and obesity. Osteoporosis is a condition that causes bones to become weaker, increasing their vulnerability to fractures, especially in the hip and wrist. Bursitis is the inflammation of a bursa, which is a fluid-filled sac located beneath the skin over joints and tendons.

Common symptoms encompass discomfort, sensitivity, inflammation, or erythema, as well as pain upon movement or palpation of the affected region. Many inflammatory mediators and miRNAs play a critical role in the underlying pathophysiology of arthritis [1–5]. The treatment approach varies according on the severity of the illness, ranging from self-care at home to surgical intervention. Orthopedic physicians may prescribe orthopedic physical therapy to enhance daily mobility. In addition, Vitamin D has been reported to attenuate inflammation, fatty infiltration and cartilage loss [6].

Orthopedic problems often manifest with dermatologic symptoms that require immediate identification to ensure appropriate referral for definitive therapy and to prevent superfluous diagnostic procedures. Therefore, it is crucial to provide particular focus to the presentations of these disorders. The purpose of this review is to identify orthopedic illnesses that have dermatologic manifestations and to explain the diagnosis of these conditions. A thorough literature search was performed during this review. Overall, a comprehensive analysis was conducted on orthopedic disorders and their corresponding disorder categories that exhibit dermatologic manifestations. Different diagnostic techniques are needed for the identified orthopedic diseases.

Orthopedic diseases frequently manifest as dermatologic manifestations. Although dermatologists often come across orthopedic conditions like ganglion cysts, diseases like carpal tunnel syndrome, while acknowledged, seldom manifest with skin issues as the initial symptom. Simultaneously, conditions like nostalgia paresthetica and brachioradial pruritus lack comprehensive characterization and are rarely discussed, particularly in regular dermatologic practice [7]. Identifying the orthopedic causes is frequently required to effectively investigate and treat these dermatologic conditions.

## 2. Literature Search Methods

An exhaustive literature search was performed, specifically targeting orthopedic diseases that manifest with dermatologic symptoms. The primary search of the relevant literature was conducted using the key words ‘orthopedic pathology’ and ‘dermatological manifestation’. The specific terms utilized for the search included ‘contracture’, ‘mass,’ ‘neuropathic itch’, ‘rash’, ‘subcutaneous nodule,’ ‘surgery,’ and ‘undifferentiated rash’.

The compilation of literature aimed to provide novel perspectives on both prevalent and rare illnesses. The study primarily examined case reports and case series that documented unusual and uncommon manifestations of orthopedic conditions, with a particular emphasis on dermatologic presentations that are infrequently observed, as well as the more normal clinical circumstances.

This research only focused on disorders that have a clearly identifiable cause related to the musculoskeletal system and particular skin symptoms. The findings are categorized based on the dermatologic presentation and the specific anatomical location where the pathology is observed. This enhances the ease of navigation.

## 3. General Manifestations

### 3.1 Psoriatic arthritis

Psoriasis is an inflammatory cutaneous disease that results in scaly, erythematous plaques that reoccur chronically [8]. Psoriatic arthritis (PsA) is an inflammatory arthropathy that results from increased bone resorption and is present in approximately 30% of psoriasis cases [8,9]. While PsA is a specific condition of psoriasis, PsA may present before psoriatic skin lesions appear in some cases [10]. Psoriasis and psoriatic arthritis are associated with osteopenia, osteoporosis, and, consequently, pathological fractures [11]. This is evident as osteoclast precursors formation is promoted by the pro-inflammatory factors of psoriasis, such as interleukin-17 (IL-17) and tumor necrosis factor alpha (TNF-α) [8,12,13]. This imposes challenges on treatment which may lead to negative outcomes necessitating surgical revision. For the best outcomes, proper considerations must be taken regarding the potential benefits of surgery; if proceeding, preoperative workup must occur prior to proceeding with surgical intervention.

Current literature suggests postponing or avoiding any surgery that could occur on psoriatic plaques as there is a higher risk of surgical site infection (SSI) and delayed wound healing due to increased bacterial load and inflammation of the area, respectively [14,15]. There has been a recent increase in popularity of perioperatively administering disease modifying antirheumatic drugs (DMARDs) such as TNF-inhibitors (TNFi) and TNF blocking agents for several weeks prior to operations to decrease inflammation associated with psoriasis, PsA, or other related conditions [16]. Due to the role of TNF in the healing cascade and inflammatory response, there is a disputed risk of delayed wound healing and increased susceptibility to SSI. In the case of spinal fusion surgery, a higher rate of reoperation was observed in TNFi patients compared to other DMARDs [17]. Meanwhile, another study demonstrated perioperative TNFi to have no significant increase in risk for SSI following total hip arthroplasty [18]. A recent systemic review demonstrated no significant increase in SSI or delayed wound healing following a variety of orthopedic operations in patients who continued DMARDs perioperatively, including TNFis [19]. While there appears to be some conflicting data in the current literature, surgeons must weigh the risks of disease flare, infection, and delayed wound healing, and collaborate with patients and rheumatologists to ensure optimal surgical outcomes.

### 3.2 Osteomyelitis

According to Marchalik et al. [20], osteomyelitis is a disorder that occurs when a certain infection affects the bone, causing it to become inflamed and damaged. Resembling cellulitis, acute osteomyelitis can be encountered in dermatological clinics, even though it is observed less commonly as the primary form of early manifestation. When dealing with a localized skin infection that does not respond to early antibiotic therapy, it is essential to consider the potential that this infection is caused by certain bacteria. When a patient has a diabetic foot ulcer that is deep and has been present for a week or longer, osteomyelitis may be suspected. This is especially true if the ulcer is positioned over a bony prominence or if there is exposed bone around the ulcer [21]. Cases of osteomyelitis brought on by nail biting are uncommonly described in the field of dermatology. Additionally, osteomyelitis can be a component of the disease known as synovitis, acne, pustulosis, hyperostosis, and osteitis (SAPHO) [22].

Regardless of the way the information is delivered, the definitive diagnosis of osteomyelitis can be identified through the process of cultivating the infectious organism that was obtained from a sterile bone sample. Osteomyelitis is typically connected with Staphylococcus aureus; however, other organisms may also be responsible for the condition, depending on the disease and the epidemiological circumstances. An increased number of neutrophils and the presence of osteonecrosis are among the findings of the histological investigation of tissue samples. When determining a diagnosis, it is possible to take into consideration several additional factors in addition to the biopsy and culture being performed. Positive blood cultures, clinical symptoms, non-specific laboratory values (such as an increased erythrocyte sedimentation rate, C-reactive protein, or white blood cell count), and diagnostic imaging techniques (particularly magnetic resonance imaging) are all potential sources of support for the diagnosis. It is advised that magnetic resonance imaging (MRI) be used as the best imaging modality for diabetic ulcers. On the other hand, the diagnosis can also be made, and the empirical therapy can be started if the bone is exposed in the ulcer bed or if it can be penetrated with a sterile surgical probe. A multidisciplinary strategy is often required for the care of osteomyelitis, which involves the collaboration of an infectious disease specialist and an orthopedic surgeon [23]. However, this is not always the case. There is a common recommendation for both antibiotic therapy and surgical debridement. In many instances, the duration of antimicrobial treatment is prolonged, and intravenous therapy may be required in specific circumstances. The removal of damaged bone and the implementation of reconstructive surgery, where necessary, are often included in the aggressive surgical intervention that is performed.

## 4. Regional Manifestations

### 4.1 Neuropathic itch

According to the findings of Weisshaar and Dalgard [24], pruritus is the symptom that is reported the most frequently in dermatology. It is estimated that almost seven percent of the population is affected by chronic itching, which is the skin condition that is reported the most frequently. On the other hand, there are other populations that might have a greater frequency of nearly 18%. According to Apfelbacher et al. [25], studies that rely on questionnaires tend to underestimate the true incidence of chronic itching. This is something that should be taken into consideration. For the most part, the evolution of chronic pruritus, which is a disease that is marked by persistent itching, is often drawn out and complicated. According to Matterne et al. [26], it is often difficult to treat, which results in a decline in the overall quality of life for persons who are afflicted because of the condition. Bernhard’s nine primary classifications [27] include neurogenic and neuropathic itch, which is a source of itching that is frequently overlooked. However, out of the nine, six are the main classes. Brain cancer, multiple sclerosis, nerve compression, and conditions such as nostalgia paresthetica (NT) and brachioradial pruritus (BRP) are the major clinical conditions [27]. These illnesses impact both the central nervous system (CNS) and the peripheral nervous system (PNS) and will be discussed in the following sections.

### 4.2 Nostalgia paresthetica

Neuropathic pruritus, also known as hereditary localized pruritus and subscapular pruritus, is categorized as a persistent sensory nerve disorder. The syndrome typically manifests as a persistent itch, often accompanied by a sensation of burning pain, numbness, and heightened sensitivity in the T2-T6 region of the back. It is believed to be caused by the compression of the posterior rami of the T2-T6 nerve roots [28]. Akram [29] has suggested that degenerative cervical spine disease is also associated with neurotrophic (NT) changes in the appropriate dermatomes. Upon examination, a hyperpigmented patch can be observed over the affected area, which is believed to be caused by scratching of the pruritic area. The syndrome has histopathological characteristics like post-inflammatory hyperpigmentation, accompanied by equivocal results from neurological tests and indications of spinal impingement due to vertebral degeneration [30].

Although frequently mistaken for cutaneous amyloidosis, histopathological examination of the hyperpigmented lesion does not reveal any amyloid deposits. Neurofibromatosis (NT) can occur in any demographic; however, it predominantly impacts individuals in their middle age, with a higher prevalence among women compared to men. A potential association with multiple endocrine neoplasia type II has been suggested. Several treatment methods have been suggested for NT, including transcutaneous electrical nerve stimulation, topical capsaicin, gabapentin, oxcarbazepine, local anesthetic nerve block, botulinum toxin, narrow-band UVB, and nerve decompression. A study by Crevits [31] found that transcutaneous electrical nerve stimulation provided partial relief. Additionally, Williams et al. [32] reported a case in 2009 where complete symptomatic relief was achieved through nerve decompression. Due to this recent advancement in treatment methods, it is crucial to have an accurate diagnosis and referral to offer a potentially curative intervention.

### 4.3 Brachioradial pruritus

According to Crevits [31], the most common symptom of BRP is a persistent itching sensation that occurs over the dorsolateral part of the upper arm or the proximal lateral forearm. According to Wallengren and Dahlback [33], the illness can manifest itself in people of any age or gender, and it has been recorded rather frequently in tropical climates that have prolonged exposure to ultraviolet light. Even though BRP is frequently described in isolation, it has recently been proved to occur in a family form, with five sisters and a brother being affected [34]. There have been several hypotheses put forward on the causes of BRP, including the possibility that UV radiation and trauma are contributing factors. On the other hand, it is commonly believed that cervical spinal illness is involved in the pathogenesis of BRP. Furthermore, it has been proposed that a significant number of instances of BRP are the result of a compression of the C5-C8 cervical nerve roots [35]. BRP has been demonstrated to be caused by a combination of cervical radiculopathy and cervical disk herniation, both of which have been validated by imaging diagnostics. It is important to highlight that the administration of ice to the pruritic area that was affected by cold-induced numbness demonstrated complete alleviation of the symptoms and could, as a result, be utilized as a diagnostic modality for BRP [36]. Other treatments that have been shown to be effective in the alleviation of pruritus caused by BRP include oral gabapentin [37], topical capsaicin, carbamazepine, and anti-inflammatory analgesics. Additionally, orthopedic treatments such as ventral cage fusion with cage implantation, cervical spine manipulation, and surgical removal of the cervical rib have also been shown to be effective.

### 4.4 Ganglion Cyst

The dermatologist is typically the first person to notice a ganglion cyst because it is the most frequent soft tissue mass of the hand and accounts for between fifty and seventy percent of all diagnoses. Women in their third, fourth, and fifth decades of life are more likely to be affected by ganglion cysts than men. The presentation is characterized by subcutaneous nodules that are soft, rubbery, compressible, and display transilluminating characteristics. These nodules are most frequently discovered on the dorsal part of the wrist, and they are attached to the tissue beneath them. Most pediatric cases exhibit volar lesions. There is a small number of dorsal wrist cysts that show with pain and neurologic symptoms, but in general, they do not cause any symptoms. There have also been reports of intraneural invasion, which has been associated with substantial disability. In rare cases, pain can be induced by doing a deep palpation across the dorsal scapholunate region or by extending the wrist to its maximum extent. According to Tuygun et al. [38], this is thought to be the result of compression of the terminal branch of the dorsal interosseous nerve that is in the wrist capsule.

The histopathological examination reveals that ganglion cysts are collections of connective tissue mucin that are well encapsulated. The genesis of ganglion cysts is not fully understood; nevertheless, there are a few probable explanations for their development, including irritation and the formation of a pseudocapsule, which can be caused by herniation or leakage of synovial tissue and fluid. Additionally, mucinous degeneration and microtrauma have been suggested as potential causes of the same condition. There have been reports of multiple ganglion cysts forming at multiple sites, which has led to the hypothesis that there is a genetic vulnerability to the development of this disorder [39].

Magnetic resonance imaging (MRI) with high signal intensity T2-weighted images is the gold standard for the examination of ganglion cysts. The diagnostic value of ultrasound has also been demonstrated. Aspiration of the cyst, which results in the production of gelatinous material, is utilized for diagnostic confirmation. According to Plate et al. [40], intervention is typically not required because most cysts in the body are asymptomatic and have been demonstrated to dissolve on their own in fifty percent of cases. On the other hand, treatment is required when the cyst causes symptoms or when it disrupts the patient’s ability to operate normally. Sclerotherapy, arthroscopic resection and puncture, and evacuation are some of the novel techniques that are now being tested in clinical trials. There have been suggestions made regarding the utilization of aspiration and intralesional corticosteroid injection; however, the effectiveness of these procedures is still debatable and need to be avoided in instances with mucous cysts. In the treatment of symptomatic cysts, resection continues to be the most effective method. It has been demonstrated that the post-resection recurrence rate for dorsal lesions is 5%, whereas the rate for volar lesions is 7%. To differentiate these lesions from the more dangerous minute synovial sarcomas, special care is essential. This is because early intervention may eliminate the requirement for harsh therapy such as amputation [41].

### 4.5 Muscle Herniation

The herniation of the tibialis anterior muscle is not an uncommon occurrence; nevertheless, it is not frequently recorded in the medical literature [42]. While the tibialis anterior muscle is commonly regarded as the primary cause of lower extremity herniation, it is important to note that other muscles have also been linked to this problem. Deficit in the fascial layer is often the cause of this herniation, which occurs very frequently. Nodules that are flesh-colored and compressible, which form gradually and do not exhibit any symptoms, are the presentation characteristics.

It is common for the subcutaneous nodules to become indistinguishable or become smaller throughout the process of muscular contraction; nonetheless, there have been occasions in which an increase in size has been seen during the process of contraction. It is possible that a biopsy needs to be performed to differentiate the herniation from other types of soft tissue tumors, such as lipomas, leiomyomas, schwannomas, and dermatofibromas. On the other hand, in contrast to muscle herniations, these lesions do not display positional variability. When this is taken into consideration, dynamic ultrasonography is still a tool for first examination that is moderately sensitive, generally available, and cost-effective. It is recommended that this method be utilized whenever there is a suspicion of a muscle herniation. A tibialis anterior muscle herniation is a rare ailment that dermatologists do not typically come across in their practice. In the literature of orthopedics and radiology, many of the cases of this ailment that have been recorded may be found. Due to the existence of a subcutaneous lump that is caused by herniation of the tibialis anterior muscle, patients frequently seek the initial evaluation of dermatologists instead of other medical professionals. Identification in a timely manner and referral to surgical care as soon as possible continue to be critical. In the present moment, there is an ongoing discussion over the management of muscle herniation in the lower extremities. Because surgery can result in compartment syndrome, also known as anterior tibial syndrome, which can have severe neurological and musculoskeletal repercussions [43], asymptomatic hernias normally do not require treatment. This is because surgery can lead to compartment syndrome.

### 4.6 Solitary enchondroma of the distal phalanx

Although it is not very usual to find enchondroma in the distal phalanx, it is the most prevalent type of primary tumor that can be detected in the hand. It is possible for people of any age to develop enchondroma; however, many solitary distal phalangeal lesions are seen in people in their thirties, and these lesions are typically asymptomatic [44]. A pathologic fracture, which happens in approximately one-third of cases, or the observation of symptoms such as clubbing, nail dystrophy, or an extended distal phalanx may prompt patients to seek medical attention at the clinic [44]. Patients may also seek medical attention at the clinic because of the presence of these symptoms. By employing lateral and oblique radiography, which typically reveals a circular, centrally positioned radiolucent abnormality, enchondromas can be identified from subungual exostoses and other bone malignancies. This is possible because enchondromas are radiolucent. Clusters of hyaline cartilage are found in the histologic examination, along with occasional well-supplied connective tissue. Additionally, there are chondrocytes present, which appear to be normal. When the size of a tumor increases rapidly or when it starts to cause pain, it is essential to evaluate the possibility that the tumor is cancerous. In extremely rare instances, Nelson et al. [45] have documented instances in which tumors have developed into chondrosarcoma, a special kind of cancer. In situations where the tumor continues to cause symptoms, when there is a loss in cortical strength, or when there is a recurrence of fracture, surgical intervention is required. According to Jurik et al. [46], the surgical therapies that are advised include curettage alone or curettage combined with cancellous bone grafting.

### 4.7 Baker’s cyst

Chatzopoulos and colleagues [47] noticed that knee arthritis frequently results in the development of Baker’s cyst. In most cases, individuals who are affected by Baker’s cysts have a palpable and varied enlargement in the popliteal region. This enlargement is often accompanied by discomfort that is exacerbated by physical exertion. When the knee is extended, the symptoms become more severe. This can be linked to the valve-like mechanism that prevents fluid from fleeing the cyst area and entering the joint. When the knee is fully extended, the symptoms become more severe. The change in size of a cyst that occurs when the joint is bent or straightened is referred to as the Foucher sign, and it is an observation that is both characteristic and definite.

The popliteal fossa, which is an extension of the knee joint, is the typical location of a Baker’s cyst, which is an enlargement of the bursa. In most cases, it manifests itself in the semimembranosus of the gastrocnemius. In addition to being actual cysts, Baker’s cysts are characterized by a synovium-based lining. In addition to rheumatologic illnesses such as rheumatoid arthritis (RA) and systemic lupus erythematosus (SLE), trauma can also be a contributing factor in the development of osteoarthritis of the knee or knee joint. Additionally, the development of knee osteoarthritis can be influenced by systemic conditions such as hypothyroidism and sarcoidosis, which are both examples of such conditions. This condition, known as Baker’s cysts, is frequently seen in both adults and children. In a clinical setting, fast diagnosis of Baker’s cyst can be accomplished using ultrasonography, which is a straightforward and highly accurate procedure. Incorrectly performing a biopsy can result in the introduction of infection into the cyst cavity, which presents a significant risk. Because of this, it is not recommended to do a biopsy prior to imaging. To evaluate the affected area, drain fluid from it, and maybe perform surgery on it, a referral to a rheumatologist and an orthopedist is required. This will result in a recurrence rate that is lower than 5%. A prolonged delay between the onset of initial symptoms and the initiation of treatment can result in significant complications. These complications include a partial blockage of the popliteal artery, which can lead to an inadequate blood supply to the lower leg (ischemia). Additionally, swelling in the lower leg and ankle can occur because of compression of the veins by an expanding cyst, which can mimic the symptoms of deep vein thrombosis. According to Woods and Sellon [48], the rupture of popliteal cysts can result in issues such as the entrapment of the posterior tibial nerve, which can result in experiencing discomfort and a reduction in feeling.

### 4.8 Soft tissue tumors of the popliteal fossa

The popliteal fossa is another location where neoplastic tumors can be discovered, including lipoma, liposarcoma, chondrolipoma, and lipoleiomyosarcoma. Mesenehymomas are the tumors that often originate from a combination of numerous different cell types. Based on the findings of Folpe and Weiss [49], a dedifferentiation that leads to malignant transformation has the potential to result in a more aggressive and difficult tumor if it is not treated promptly. Chondrolipomas that are seen in the popliteal fossa are characterized by the presence of subcutaneous nodules that are painless. According to Ohtsuka [50], the size of these nodules can range from less than one centimeter to lesions that have been measured to be as large as ten centimeters above the surface. There is also the possibility that lesions are present simultaneously in other parts of the body. An excision using surgical means is the treatment of choice. Nevertheless, popliteal sarcomas provide considerable obstacles in terms of treatment, and it may be necessary to amputate the limbs that are afflicted by the disease [51].

### 4.9 Piezogenic pedal papules

Piezogenic pedal papules, often known as PPP, are a disorder that is encountered quite commonly in the general population. According to study, the frequency of PPP in children can reach as high as 72 percent [52]. The term “presentation” refers to the emergence of several small, raised skin lesions that are referred to as papules. The size of these papules might vary depending on the age of the individual as well as the amount of physical activity they engage in. The lesions can be found on the wrists or the heels, with the inner side of the sole of the heel being the most prevalent location for pediatric patients [53]. When weight is placed on the heel and pressure is applied to the wrist, the papules become visible on the skin. There is a different manifestation of the lesions in pediatric patients, which is defined by the existence of nodules rather than papules and the absence of a piezogenic component [54]. Both characteristics are present.

In most cases, the papules do not create any symptoms; nonetheless, they may cause pain, which greatly hinders the patient’s capacity to participate in activities. It is considered that the infiltration of fat via the dermis is a contributing factor in the development of the lesions. A possible relationship between PPP and the occurrence of connective tissue illnesses, such as Ehlers-Danlos syndrome, has been suggested as a possible reason for this association. According to Cho et al. [55], there has been no successful identification of a definite genetic relationship to PPP. An indication that connective tissue weakness may be present in certain patients is the fact that there have been cases of simultaneous herniation of the tibialis anterior muscle. Ultrasonography provides the practitioner with a quick and convenient confirmation test that can be conducted in the office. This contrasts with the traditional method of providing a definite diagnosis from a complete history and physical examination, which typically results in typical findings. When it comes to athletes, this is especially beneficial because keratosis could conceal the papules. In most instances, the treatment is typically non-invasive; nevertheless, surgery may be considered for painful lesions or for cosmetic considerations. Under these circumstances, it is advisable to refer the patient to an orthopedic surgeon for further evaluation. According to Marchalik et al. [20], electro-acupuncture has been demonstrated to be effective in the management of painful papule infections.

### 4.10 Fibromatosis

There are a variety of conditions that fall under the umbrella of digital fibromatosis. These conditions include infantile digital fibromatosis, inclusion body fibromatosis, Reye tumor, and recurring digital fibrous tumor of childhood. Digital fibromatosis is characterized by the appearance of subcutaneous nodules that are red or pink in color and can range in size from one centimeter to two centimeters [56,57]. Lesions of this nature often appear on the extensor surfaces of the first four fingers and toes, excluding the thumb and the big toe from their scope of manifestation. They are secure in their attachment to the tissues that lie beneath them. It is possible for certain subtypes to demonstrate themselves on the palms and soles of the extremities. One histologic subtype of myofibromatosis, known as infantile myofibromatosis, often occurs in eighty percent of patients before the age of two years. In accordance with the findings of Montgomery et al. [58], it is possible to identify it by the presence of lesions in the viscera and bone.

## 5. Treatment Related Manifestations

### 5.1 Koebner phenomenon

The Koebner phenomenon (KP) describes the appearance of skin lesions consistent with a patient’s existing cutaneous disease on previously unaffected skin following trauma, such as at the site of surgery or injury [59,60]. It is important to note that experimentation has found that not all types of trauma result in KP nor in the same regions in the same patient, and thus, can present with several manifestations following orthopedic treatment [61]. True Koebnerization manifestations include psoriasis, linear lichen planus, and vitiligo [59]. This review focuses solely on true Koebnerization manifestations following orthopedic treatment including surgery and other therapeutic interventions.

### 5.2 Koebner phenomenon in psoriasis

In 20% of psoriasis patients, the Koebner phenomenon is present and leads to psoriatic lesions following irritation or trauma, such as at the surgical site [62]. Like PsA, KP-induced psoriasis presents a similar negative risk of outcomes following surgical intervention, including delayed healing and SSI. According to one systemic review, however, some retrospective studies have found increased risk of post-operative complications, while others have found no such risk with similar cohorts [63]. While the literature on KP psoriasis is limited, surgeons must still collaborate with patients and assess possible outcomes to determine if surgical intervention is appropriate. The benefits of perioperative use of DMARDs for KP psoriasis patients has not been directly studied; like PsA, further investigation should be conducted to ensure a decrease in post-operative lesions and that the potential risk of delayed healing and infection is minimal.

### 5.3 Linear lichen planus

Lichen planus (LP) is defined as a chronic inflammatory and immune-mediated disease that affects the skin with lesions characterized as polygonal, flat-topped, violaceous papules and plaques with overlaying white scales [64]. LP also exhibits the Koebner phenomenon following skin trauma such as at the site of orthopedic trauma or surgical incisions [65]. Pertaining specifically to surgeries, linear lichen planus (LLP) is seen in less than 0.2% of all LP patients [66]. These lesions typically following Blaschko lines which trace the migration of embryonic cells [67]. As of the current literature, there have been two cases reported of patients developing lichen planus due to orthopedic implants.

In the first case, a 64-year-old female patient presented with LLP that began 7 months post-operation which included placement of a chromium and cobalt-based implant following a femoral bone fracture. Though the patient refused a patch test, the authors suggest that the LLP was due to cytokine release due to hypersensitivity to the metals of the implant [67]. In their reasoning, they refer to the occurrence of oral lichen planus cases primarily present following hypersensitivity to specific metals in dental implants [68]. In the case of orthopedic implants, there is evidence to suggest there may be an underlying metal allergy cause; however, it is unclear whether these symptoms could be attributed to hypersensitivity as patch testing was declined by the patient. It is also unclear if the presence of LLP is attributed to the Koebner phenomenon, as there is no mention the patient having a history of KP.

The second case involves a young male of undisclosed age who fractured his right leg and was treated with stainless steel orthopedic implants 2 years prior to examination. He began to display LLP 15 days prior to consultation, localized entirely to the lower right leg and foot at the region of trauma/surgery, with the largest plaque corresponding to the suture line. Unlike the prior case, patch testing was conducted and negative, further ruling out potential hypersensitivity from orthopedic implant metals. The authors also reject the notion of its presence due to the Koebner phenomenon as the patient did not have prior history of LP [69].

It is important to note that, while the symptoms of these two cases occurred several months post-operation, this also does not eliminate the potential for prosthesis-induced hypersensitivity. Due to the rarity of these cases, there are no studies that provide an underlying mechanism or risk factors for LLP following orthopedic trauma; however, LLP is still a potential manifestation in cases where hypersensitivity is ruled out. Since LLP cases involving dental implantation are more prevalent, future cases may benefit from literature surrounding the treatment of oral manifestations of LLP.

### 5.4 Vitiligo

Vitiligo is another indicator attributed to the KP but is less frequently depicted in the literature in comparison to psoriasis and LLP, likely due to being primarily cosmetic. KP incidence has been reported to range from 21–62% of vitiligo patients, varying due to differing criteria and assessment methods [70]. The underlying mechanisms that contribute to this phenomenon include immune-mediated mechanisms, increased oxidative stress, defective melanocyte adhesion, and melanocyte growth factors [70].

One such case following knee replacement surgery led to vitiligo along and adjacent to the incision line with a patient with no history of eczematic allergies, attributed to increased mechanical oxidative stress [71]. The authors of the case note that metal allergies from the implant are a possible explanation, leading to immune-related mechanisms that induce vitiligo, yet do not mention a history of metal allergy [71]. Metal hypersensitivity testing in such cases would be ideal to distinguish KP-induced vitiligo from contact vitiligo.

More investigation into the underlying mechanism, prevalence, and risk factors need to be investigated in the current literature to help understand KP induced vitiligo and help surgeons determine the likelihood and potential severity in at-risk patients. Currently, KP-induced vitiligo is distinguished from general vitiligo by criteria such as being induced by repeated pressure, repeated friction, or trauma, either superficial or dermo-epidermal [70]. Delayed hypersensitivity to benzoyl peroxide found in bone cement used in the surgery was also of concern, yet a previous study did not note hypo/depigmentation as complications [72]. While vitiligo is primarily cosmetic, it is important to note the negative effect on self-esteem and confidence of patients, especially with individuals with increased melanin production where depigmentation is a more drastic change.

### 5.5 Metal allergies

Hypersensitivity to metals is not an uncommon phenomenon, reported to affect between 10 and 15% in the general population [73]. Nickel metal is a common component of metal implanted devices such as those used in orthopedic intervention. Of roughly 4000 patch-tested patients across facilities in America, one study found hypersensitivity to nickel present in roughly 18% of patients [74]. Other common metals include cobalt, vanadium, chromium, and titanium which can be found both as the sole metal in an implant or mixed with several as an alloy. Hypersensitivity to cobalt, chromium, and titanium are present but less common, yet rates may also be due to increased use of these metals in recent decades. Another major factor in orthopedic surgery is the use of bone cements, which contain components and antibiotics that can also induce hypersensitivity [75,76]. Surgeons must consider these various factors when determining a treatment approach and selecting the appropriate implant.

Complications from metal implants due to allergies may appear on the skin in the form of localized or systemic dermatitis, urticaria, and delayed/impaired wound healing [77]. Treatment is not consistent across surgical procedures or implant types, with lack of evidence for a universal protocol; instead, management based on case-by-case decisions is more common [78]. Expert panels have agreed on allowing adequate time for symptoms to subside as healing and integration of the implant improve [77].

**Figure 1:**
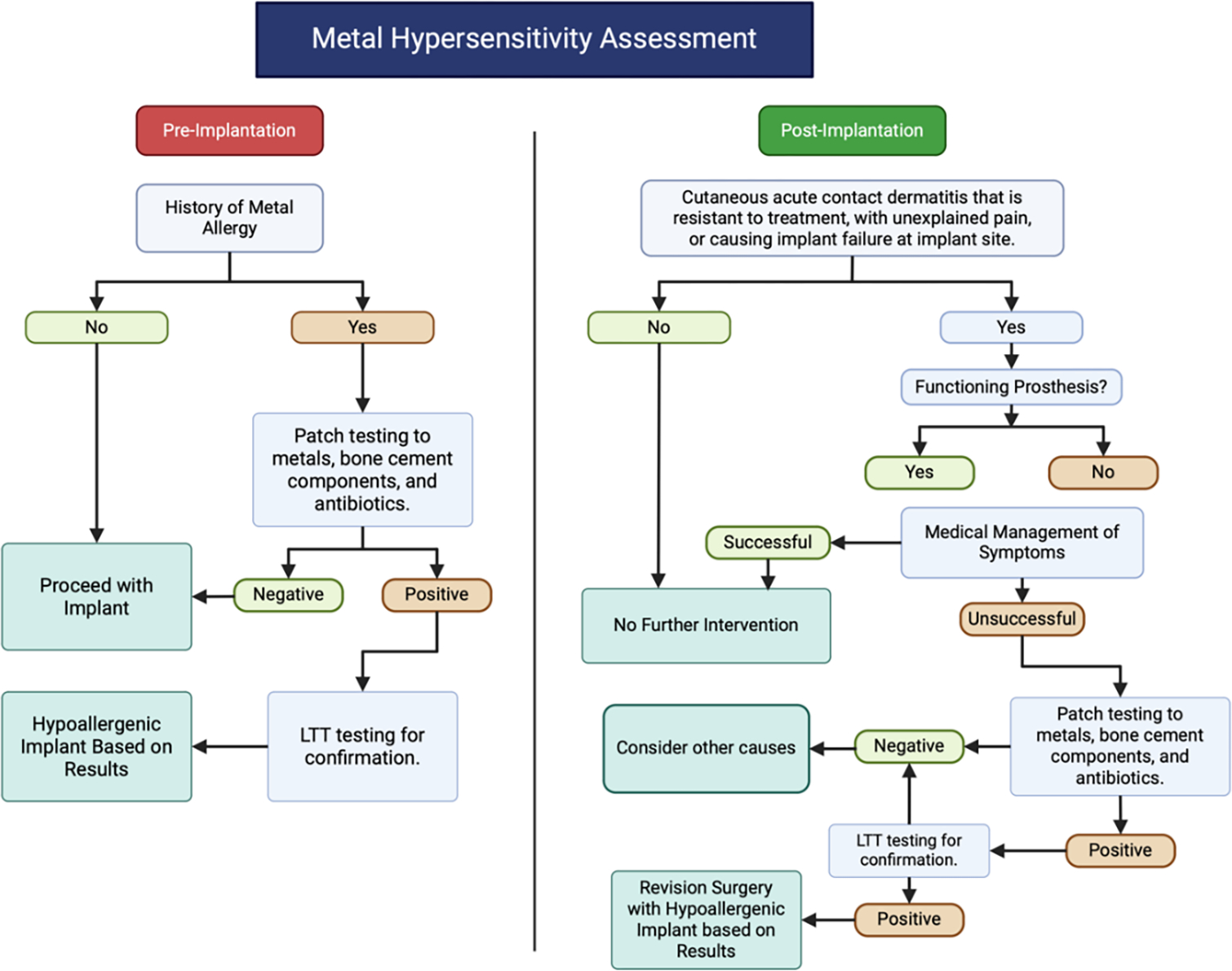
This is a flowchart for potential use when approaching metal allergy pre- and post-implantation adapted from Richards et al. [81] to include increased use of LTT.

Currently, allergy testing is typically reserved for patients with an existing history of hypersensitivity to implants, rather than those receiving their first implant [77]. Surgeons should consider asking patients if they have had contact dermatitis from wearing jewelry or watches. One study found that of patients who self-reported jewelry sensitivity, 93% patch-tested positive to at least 1 metal allergen [79]. Regarding testing modalities, patch testing has been the standard method for identifying metal allergies yet may result in inconclusive testing or induce sensitization [78]. The physician may opt for more accurate lymphocyte transformation testing (LTT), but allergens are limited and limited availability. Lack of standardization and guidelines opting for patch-testing currently limit the use of LTT in orthopedic practice [80]. Figure 1 suggests a potential workflow adapted from [81] that incorporates the use of both patch testing and LTT to determine the approach whether pre-operatively or post-operatively. While post-operative treatment of hypersensitivity varies enough to be limited to each surgeon’s judgement, guidelines must be updated to reflect the use of LTT in conjunction with patch testing to decrease the rate of complications from implants.

## 6. Conclusion

Orthopedic diseases, such as rheumatoid arthritis, osteoarthritis, osteoporosis, and bursitis, are prevalent and often manifest as dermatologic symptoms. These conditions require immediate identification to ensure appropriate referral for definitive therapy and prevent unnecessary diagnostic procedures. This review aims to identify orthopedic illnesses with dermatologic manifestations and explain their diagnosis. A thorough literature search was conducted, focusing on disorders with clearly identifiable causes related to the musculoskeletal system and specific skin symptoms.

Neuropathic itching is the most common skin condition reported in dermatology, affecting almost seven percent of the population. It is often difficult to treat and can lead to a decline in quality of life. Neurofibromatosis (NT) is a persistent sensory nerve disorder that can cause burning pain, numbness, and heightened sensitivity in the T2-T6 region of the back. Treatment methods for NT include transcutaneous electrical nerve stimulation, topical capsaicin, gabapentin, oxcarbazepine, local anesthetic nerve block, botulinum toxin, narrow-band UVB, and nerve decompression.

Brachioradial pruritus (BRP) is a rare condition that is often misdiagnosed and treated with various treatments. BRP, a persistent itching sensation, is a common symptom in people of any age and gender, often found in tropical climates with prolonged exposure to ultraviolet light. It is often a family-related condition, with five sisters and a brother affected. Causes include UV radiation, trauma, cervical spinal illness, and cervical disk herniation. Ice application has been shown to alleviate symptoms, and other treatments include oral gabapentin, topical capsaicin, carbamazepine, and anti-inflammatory analgesics. Orthopedic treatments like ventral cage fusion, cervical spine manipulation, and surgical removal of the cervical rib have also been effective.

Osteomyelitis, a disorder causing bone inflammation and damage, is a common cause of BRP. Diagnosis can be made through positive blood cultures, clinical symptoms, non-specific laboratory values, and diagnostic imaging techniques. Magnetic resonance imaging (MRI) is recommended for diagnosing diabetic ulcers. A multidisciplinary approach is often required, involving antibiotic therapy and surgical debridement. In some cases, intravenous therapy may be required.

Ganglion cysts are soft tissue masses in the hand, affecting women in their third, fourth, and fifth decades. They are characterized by soft, rubbery, compressible subcutaneous nodules on the dorsal part of the wrist. The genesis of ganglion cysts is not fully understood, but they may be caused by irritation, pseudocapsule formation, mucinous degeneration, microtrauma, or genetic vulnerability. Magnetic resonance imaging (MRI) and ultrasound are the gold standard for examining ganglion cysts. Treatment is typically not required, but resection is the most effective method for symptomatic cysts. Muscle herniation is a rare condition, often caused by a lack in the fascial layer. Dynamic ultrasonography is a moderately sensitive and cost-effective tool for first examination. Early identification and referral to surgical care are crucial for managing muscle herniation in the lower extremities.

Enchondroma, a solitary primary tumor in the distal phalanx, is the most common type of hand lesions, typically asymptomatic in the thirties. Symptoms include clubbing, nail dystrophy, and an extended distal phalanx. Enchondromas can be identified using radiography and can cause pain or cancer. In rare cases, tumors can develop into chondrosarcoma. Surgical intervention is required for cases causing symptoms, loss of cortical strength, or recurrence of fractures. Baker’s cysts, an enlargement of the bursa in the knee joint, are often caused by knee arthritis and can be caused by rheumatologic illnesses, trauma, and systemic conditions.

Soft Tissue Tumors popliteal fossa, neoplastic tumors, and piezogenic pedal papules are common conditions in the general population. Mesenehymomas, characterized by painless subcutaneous nodules, can lead to aggressive and difficult tumors if not treated promptly. Piezogenic pedal papules (PPP) are small, raised skin lesions found on wrists or heels, with pediatric patients experiencing a different manifestation. Ultrasonography provides a quick and convenient diagnosis, but surgery may be considered for painful lesions or cosmetic considerations. Digital fibromatosis, a type of fibromatosis, is characterized by subcutaneous nodules red or pink in color and can range in size from one centimeter to two centimeters. Infantile myofibromatosis, a subtype of myofibromatosis, often occurs in patients before the age of two years.

While many dermatological manifestations must be considered prior to treatment, orthopedic treatments and surgeries themselves are prone to manifestations including the Koebner Phenomenon and metal hypersensitivity. KP can manifest as psoriasis, LLP, and vitiligo. Psoriasis patients may experience KP-induced psoriatic lesions following trauma or surgery, leading to negative outcomes such as delayed healing and surgical site infections. However, the literature on KP psoriasis is limited, and further research is needed to determine the benefits of perioperative use of disease modifying anti-rheumatic drugs and minimize post-operative complications. KP-induced LLP and vitiligo, although rarer, has been reported in orthopedic patients, and its underlying mechanism and risk factors require further investigation. Metal allergies to various metals in implants continue to contribute to complications in orthopedic surgery, leading to dermatitis, urticaria, and impaired wound healing. Proper allergy testing, including patch testing and lymphocyte transformation testing, following a universal protocol should be considered to minimize the risk of complications from metal implants. Overall, a better understanding of KP and metal allergies is crucial for surgeons to make informed decisions and improve patient outcomes in orthopedic interventions.

**Figure 2:**
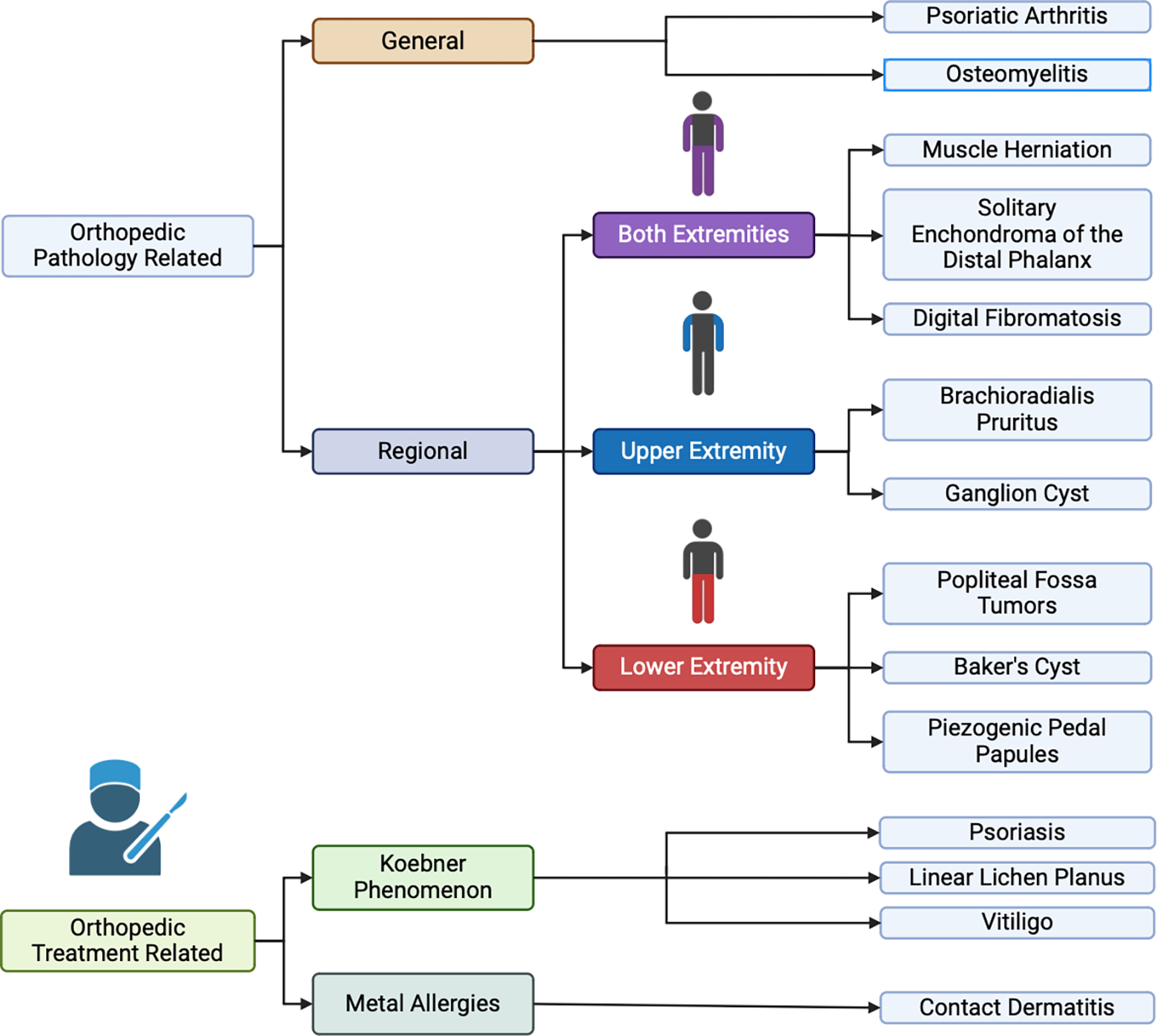
This is a flowchart showing various pathologies discussed in this article, divided initially by manifestation prior to and post treatment. The orthopedic pathology related manifestations are divided into general and regional, with regional subdivided into the extremities. KP related manifestations and metal allergies are separate orthopedic treatment related categories.

Dermatopathies should be viewed not only as obstacles but also as valuable risk factors for orthopedic surgeons when planning and executing treatments. As illustrated in Figure 2, most of these manifestations can present prior to treatment in different regions of the body; though individual manifestations, some of these can present concurrently and may be misidentified for one another. These warning signs can offer valuable insights into how the patient’s body may respond to surgery. In instances where dermatopathies manifest after treatment, it is crucial to assess whether these reactions are related to infections or other factors. By carefully evaluating and considering these warning signs, surgeons can better anticipate and address potential complications, ultimately improving patient outcomes.
